# Case Report: Simultaneous repair of cerebrospinal fluid otorrhea and cochlear implantation in a patient with single-sided deafness and inner ear anomaly

**DOI:** 10.3389/fsurg.2026.1757460

**Published:** 2026-03-30

**Authors:** Sen Chen, Huamao Cheng

**Affiliations:** Department of Otorhinolaryngology, Union Hospital, Tongji Medical College, Huazhong University of Science and Technology, Wuhan, China

**Keywords:** cerebrospinal fluid otorrhea, cochlear implant, incomplete partition type I, inner ear anomaly, unilateral deafness

## Abstract

An-five years old male presented with recurrent attacks of headache and clear nasal discharge. Magnetic resonance cerebrospinal fluid (CSF) imaging demonstrated descent of the cerebellar tonsils, with CSF signal identified within the left middle ear. Temporal bone computed tomography showed the left cochlea was incomplete partition type I. Audiological evaluation revealed profound sensorineural hearing loss in the left ear of the pediatric patient. The surgical approach of mastoidotomy with exploration was utilized in the management of this pediatric case. CSF otorrhea was confirmed and the CSF leak was successfully localized at the oval window. The pediatric patient underwent repair of CSF otorrhea with concurrent cochlear implantation. Postoperatively, there was no recurrence of CSF otorrhea, and hearing was restored in the left ear. This report describes the first case of simultaneous repair of cerebrospinal fluid (CSF) otorrhea and cochlear implantation for the management of both CSF otorrhea and acquired unilateral profound sensorineural hearing loss.

## 1 Introduction

Cerebrospinal fluid (CSF) otorrhea is defined as the discharge of cerebrospinal fluid through the ear, which predominantly results from trauma, congenital malformations, or iatrogenic causes. Spontaneous CSF (sCSF) otorrhea, which accounts for approximately 23.9%–54% of all cases, is defined by an abnormal communication between the subarachnoid space and the middle ear, primarily due to congenital inner ear dysplasia (1). Although often clinically silent without a history of trauma or meningitis, spontaneous CSF otorrhea in children can present as a subtle, clear nasal discharge that is easily overlooked, which, if left untreated, can result in life-threatening meningitis (2).

Congenital malformations of the inner ear can create a conduit through the labyrinth, allowing CSF to flow into the middle ear. In children, inner ear malformations are a leading cause of sCSF otorrhea (3). These malformations include incomplete partition types, cochlear hypoplasia, cochlear aplasia, common cavity and Mondini dysplasia (4–6). Furthermore, these children often present with varying degrees of hearing loss. Thus, preserving or restoring auditory function during the repair of the CSF otorrhea poses a significant clinical challenge (7, 8).

Here, we report on the simultaneous repair of sCSF otorrhea and cochlear implantation in a pediatric patient who exhibited acquiced unilateral deafness caused by sCSF otorrhea and inner ear malformation. It is hoped that this case report will offer valuable insights for clinical decision-making in managing unilateral deafness with CSF otorrhea and contribute to the expanding body of evidence regarding cochlear implantation candidacy.

## 2 Case description

### 2.1 2.1.Case and diagnostic assessment

A 5-year-old boy, who had passed newborn hearing screening bilaterally, presented with a six-month history of recurrent clear watery rhinorrhea and headache. A decrease in hearing acuity in the left ear was noted three months ago. During this period, the child experienced an episode of fever accompanied by headache and vomiting. The symptoms were attributed to a upper respiratory tract infection and resolved promptly after antibiotic treatment in the Pediatrics Department of Union Hospital. MRI findings revealed no evidence of meningitis but demonstrated descent of the cerebellar tonsils and the presence of cerebrospinal fluid signal within the middle ear. The patient was subsequently referred to the Otorhinolaryngology Department of Union Hospital for further evaluation.

**Figure 1:**
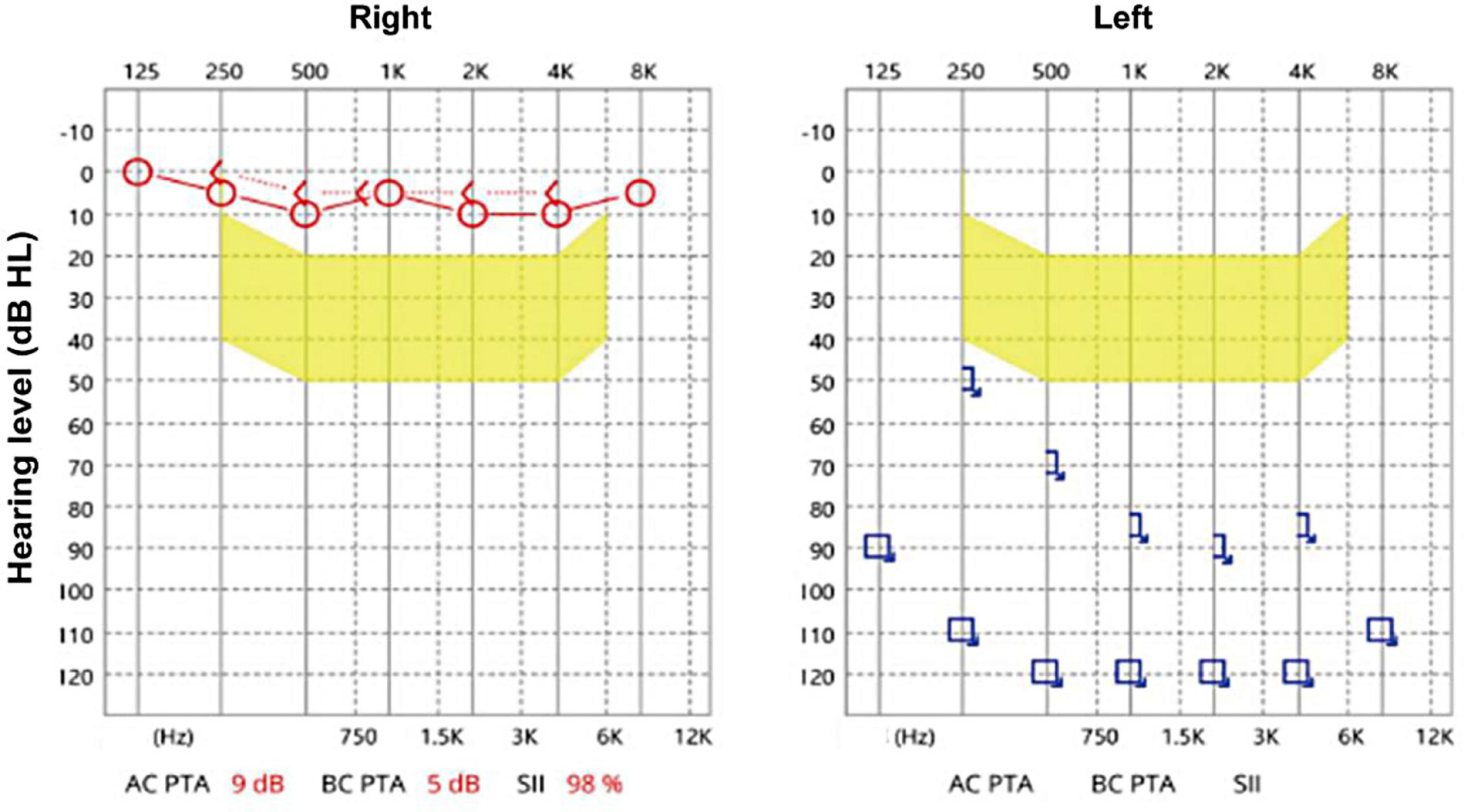
Pure-tone audiometry of this case before the operation.

**Figure 2:**
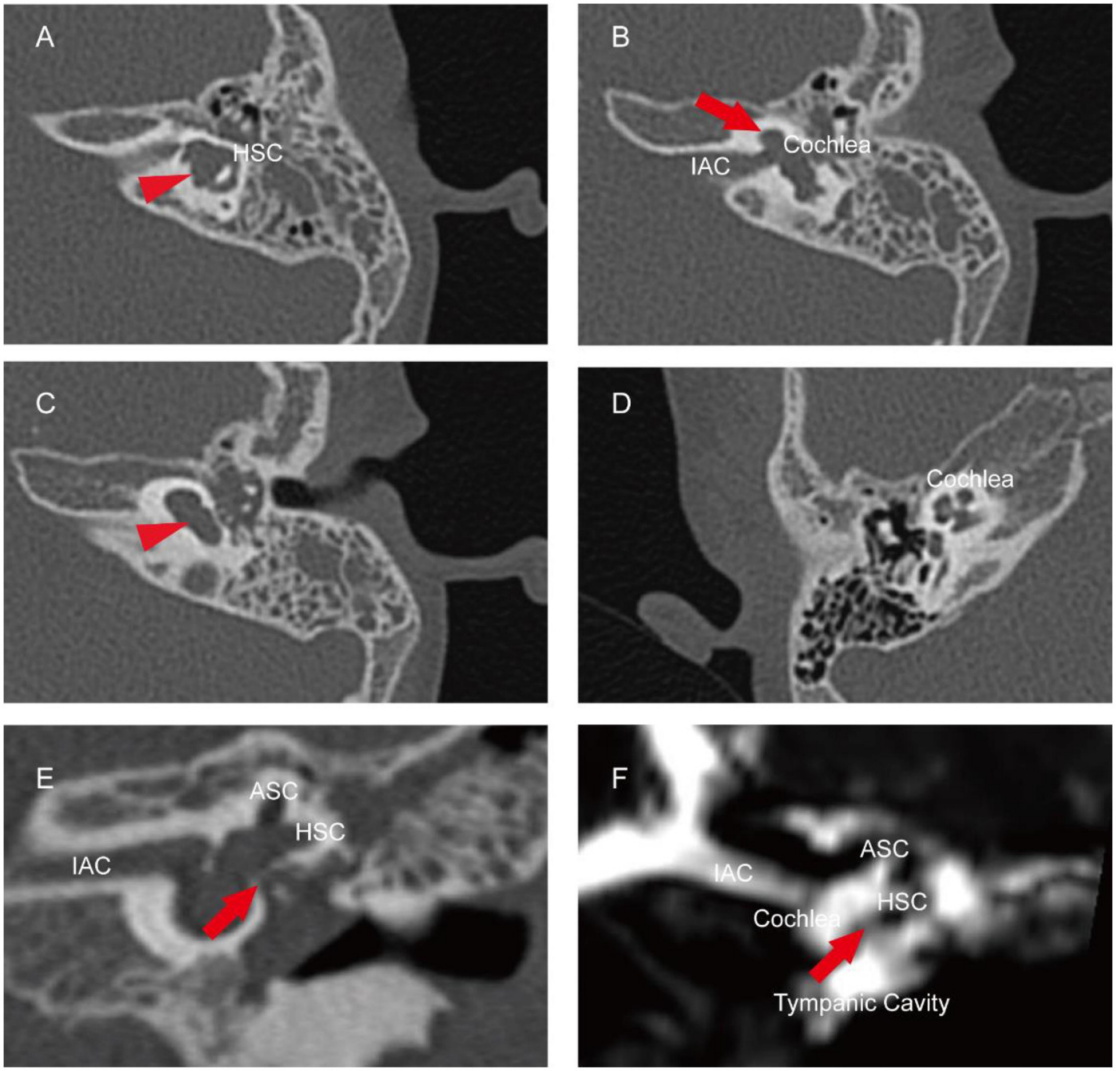
Preoperative imaging findings. **(A)** CT image at the level of the horizontal semicircular canal. The red arrowhead indicates an malformed HSC. **(B)** CT image at the level of the cochlea. The red arrow indicates the absence of the modiolus within the malformed cochlea. **(C)** The red arrowhead indicates the malformed and enlarged vestibule. **(D)** CT image of the normal right cochlea in the pediatric patient. **(E)** Coronal CT image of the tympanic cavity. The red arrow points to a bony discontinuity in the oval window region. Additionally, an isodense abnormal signal is present within the tympanic cavity. **(F)** Coronal T2-weighted MR image corresponding to panel E. The red arrow indicates an abnormal fluid communication between the cochlea and the tympanic cavity. The tympanic cavity is also filled with water signal. HSC, horizontal semicircular canal; ASC, anterior semicircular canal; IAC, internal auditory canal.

A comprehensive battery of audiological tests was administered. Pure-tone audiometry and auditory brainstem response (ABR) testing confirmed profound sensorineural hearing loss in the left ear and normal hearing in the right ear (Figure 1 and Table 1). Multiple auditory steady-state evoked response (ASSR) findings demonstrated average thresholds exceeding 85 dB nHL in the left ear. The acoustic immittance disclosed the presence of a type B curve in the left ear and a type As curve in the right ear (Table 1). High-resolution temporal bone CT imaging demonstrated the following abnormalities on the left side: a cystic cochlea without a modiolus (Figure 2B, red arrow), a dilated vestibule (Figure 2C, red arrowhead), and underdevelopment of the horizontal and posterior semicircular canals (Figure 2A, red arrowhead). Coronal views of the temporal bone CT revealed a bony defect at the stapes footplate (red arrow), with findings suggestive of a fistula (Figure 2E). On the same coronal plane of the CSF MRI, the fluid signal revealed continuity between the basal turn of the cochlea and the tympanic cavity, suggesting a fistulous tract (Figure 2F, red arrow). Based on the above results, the findings are consistent with an inner ear malformation presenting with CSF otorrhea and unilateral sensorineural hearing loss. Therefore, definitive surgical management involving repair of the CSF leak with concurrent cochlear implantation is planned.

**Table 1:** Objective audiological test results.

Side	ABR	ABR (BC)	ASSR^a^	Acoustic immittance
R	10	10	25	Type As
L	N	N	95	Type B

^a^ The arithmetic mean of the hearing thresholds at 0.5k, 1k, 2k, and 4k Hz. R, right ear; L, left ear; BC, boneconduction.

### 2.2 Surgical procedure

**Figure 3:**
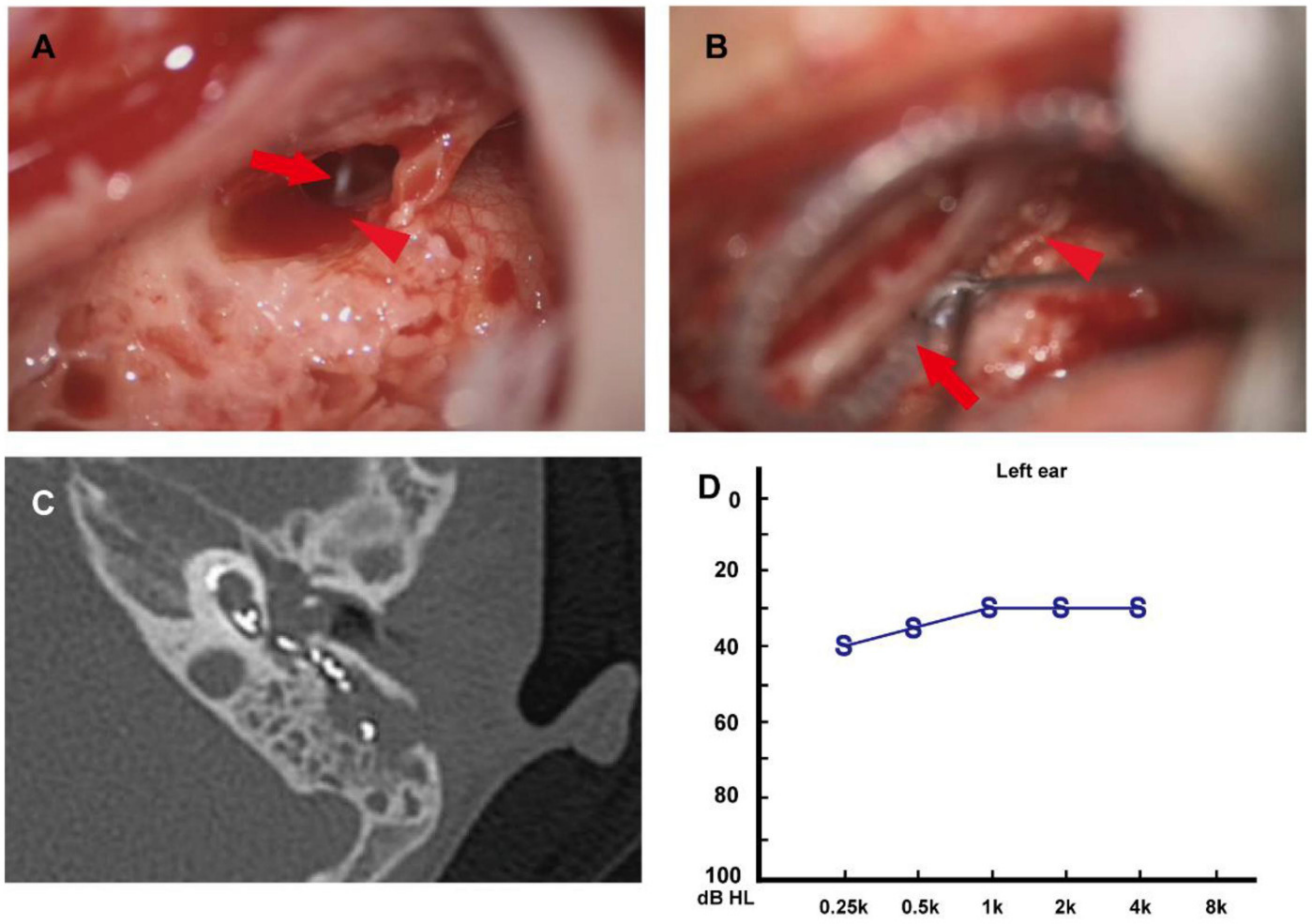
**(A)** Intraoperatively, the facial recess was opened. The red arrowhead indicates clear cerebrospinal fluid leaking around the oval window. The red arrow points to the stapedius muscle. **(B)** The cochlear electrode was implanted via the round window. The red arrowhead denotes the cessation of cerebrospinal fluid leakage after the oval window area was obliterated with muscle. The red arrow indicates the cochlear electrode. **(C)** Postoperative CT demonstrates correct intrascalar placement of the cochlear implant electrode. **(D)** The one-month postoperative sound field audiometry demonstrates the hearing results of the left ear.

The surgical approach consisted of a retroauricular “C"-shaped skin incision, via which a mastoidectomy and subsequently a posterior tympanotomy were performed. The facial recess was then opened, and a CSF fistula was identified at the oval window (Figure 3A). After the incus was removed, we carefully examined the area around the oval window and the footplate of the stapes. In this patient, the stapes footplate was extremely thin and slightly bulging outward, and a small fistulous opening was identified at the junction between the stapes footplate and the annular ligament. We prepared a strip of temporalis fascia slightly thicker in diameter than the fistulous opening. Approximately 1 mm of the muscle fascia strip was inserted into the fistula, while the remaining portion was placed around the stapes to seal the fistula. The fascia was then secured in place with surgical glue. The cochlear electrode was then implanted through a drilled round window niche, and the insertion site was packed with additional muscle to ensure a watertight closure (Figure 3B). Subsequently, the patient received a 5-day course of antibiotics after the surgery.

## 3 Results

The patient remained asymptomatic for CSF otorrhea and headache postoperatively. A follow-up CT scan one week after surgery confirmed the appropriate intracochlear position of the electrode array (Figure 3C). Sound field audiometry conducted one month postoperatively revealed an average hearing threshold of 31 dB HL in the implanted ear (Figure 3D).

## 4 Discussion

Spontaneous CSF otorrhea is a diagnosis of exclusion, characterized by a CSF leak with no identifiable cause, including prior temporal bone trauma, surgery, infection, or malignancy. In pediatric and adolescent patients, congenital inner ear malformations represent the most common cause of sCSF otorrhea (3, 7, 9, 10). CSF otorrhea resulting from an inner ear malformation necessitates a complete fistulous tract. This pathway consists of a proximal communication between the subarachnoid space and the inner ear, and a distal defect between the inner ear and the middle ear. A bony defect or abnormal enlargement at the fundus of the internal auditory canal creates a proximal communication, allowing CSF to track into the cochlea or vestibule through a dural breach. Therefore, malformations featuring these abnormalities are highly predisposed to CSF otorrhea. Several inner ear malformations, such as common cavity deformity, cochlear aplasia, and incomplete partitions, have been reported in association with CSF otorrhea, as they all share these characteristic pathogenic features (9, 11, 12). The distal fistula demonstrates a distinct predilection for the oval window (stapes footplate). The round window, promontory, and hypotympanum constitute less common sites for such defects (12–14). In this case, the patient's cochlea and vestibule were partially fused and enlarged, with an absent modiolus, which is characteristic of an Incomplete Partition Type I (IP-I) malformation. This findings is consistent with the characteristics of a proximal communication. As the stapes and its annular ligament are common vulnerable sites separating the inner and middle ear, the site of the distal fistula is often located at this position. Intraoperatively, the distal fistula was identified at the round window and its annular ligament, confirming the distal pathway. Notably, the delayed onset of both CSF otorrhea and hearing loss suggests that these communications achieved patency postnatally.

Single-sided deafness (SSD) does not universally mandate cochlear implantation. Some studies indicated that childhood SSD can lead to deficits in cognitive, speech, and language development, ultimately affecting academic and social performance (15, 16). Conversely, a separate study failed to establish a definitive negative impact of SSD on speech development and daily communication in children (17). Nevertheless, studies have found that children with SSD derive greater benefit from cochlear implantation compared to adults. These benefits are primarily manifested as improved speech recognition scores and sound localization abilities. Furthermore, a shorter duration of deafness is associated with a greater degree of benefit (16, 18).

In this case report, the child exhibited well-developed speech and the ability to communicate effectively in daily life, with clear articulation. However, there was a concern that performing CSF leak repair alone could lead to cochlear ossification, thereby precluding future cochlear implantation and resulting in the permanent loss of opportunity for binaural hearing. Additionally, the relatively recent onset of the child's unilateral deafness was also taken into consideration, as it suggested a potential for greater benefit from cochlear implantation. Following detailed counseling, the procedure was performed in collaboration with the family, with the child undergoing simultaneous CSF leak repair and cochlear implantation. Objective measures of sound localization could not be obtained due to a lack of appropriate assessment tools. During follow-up, the parents reported a subjective improvement in the child's hearing function and good tolerance to prolonged device use. The reported benefits included improved speech perception in noise and the ability to accurately identify sound sources.

## 5 Conclusion

In conclusion, the combined approach of sCSF leak repair and cochlear implantation demonstrates efficacy in managing inner ear malformations presenting with otorrhea and profound hearing loss. In children with SSD, this strategy offers a dual advantage: it definitively resolves the CSF leak while simultaneously restoring binaural auditory input, leading to improved hearing outcomes and quality of life.

## Data Availability

The original contributions presented in the study are included in the article/Supplementary Material, further inquiries can be directed to the corresponding author.
